# Diagnostic accuracy of a smartphone bedside test to assess the fixation suppression of the vestibulo-ocular reflex: when nothing else matters

**DOI:** 10.1007/s00415-020-09947-5

**Published:** 2020-07-08

**Authors:** Florin Gandor, Manfred Tesch, Hannelore Neuhauser, Doreen Gruber, Hans-Jochen Heinze, Georg Ebersbach, Thomas Lempert

**Affiliations:** 1 Movement Disorders Hospital, Kliniken Beelitz GmbH, Strasse nach Fichtenwalde 16, 14547 Beelitz-Heilstätten, Germany; 2 Department of Neurology, Otto-Von-Guericke University, Magdeburg, Germany; 3 Department of Neurology, Schlosspark-Klinik Berlin, Berlin, Germany; 4 Robert-Koch-Institut, Berlin, Germany

**Keywords:** Vestibulo-ocular reflex, Fixation suppression of the vestibulo-ocular reflex, Bedside test, Cerebellar syndrome

## Abstract

**Objective:**

Validation of a bedside test to objectify the fixation suppression of the vestibulo-ocular reflex (FS-VOR) in patients with a cerebellar syndrome and healthy controls.

**Methods:**

The vestibulo-ocular reflex and its fixation suppression were assessed by video-nystagmography (VNG) in 20 healthy subjects (mean age 56 ± 15) and 19 patients with a cerebellar syndrome (mean age 70 ± 11). The statistical cutoff delineating normal from pathological FS-VOR was determined at the 2.5th percentile of the normal distribution of the healthy cohort. VNG was then compared to a bedside test, where eye movements were recorded with a smartphone while patients were rotated on a swivel chair at a defined speed and amplitude. These videos were rated as normal or pathological FS-VOR by six blinded raters, and results compared to VNG.

**Results:**

VNG in healthy controls showed FS-VOR with a reduction of nystagmus beats by 95.0% ± 7.2 (mean ± SD). The statistical cutoff was set at 80.6%. Cerebellar patients reduced nystagmus beats by only 26.3% ± 25.1. Inter-rater agreement of the smartphone video ratings was 85%. The sensitivity of the video ratings to detect an impaired FS-VOR was 99%, its specificity 92%. Inter-test agreement was 91%.

**Conclusion:**

The smartphone bedside test is an easily performed, reliable, sensitive, specific, and inexpensive alternative for assessing FS-VOR.

**Electronic supplementary material:**

The online version of this article (10.1007/s00415-020-09947-5) contains supplementary material, which is available to authorized users.

## Introduction

Fixation suppression of the vestibulo-ocular reflex (FS-VOR) is a reliable clinical test of cerebellar function [1, 2], and its disturbance indicative of cerebellar pathology [1, 3, 4]. In movement disorders, it can serve as an additional feature to differentiate idiopathic Parkinson's disease from atypical Parkinsonian syndromes such as multiple system atrophy (MSA) [5]. The gold standard of assessing FS-VOR is utilizing a video-nystagmography (VNG) system and a motor-driven rotary chair. However, not only are such devices expensive and need expertise in interpretation, but they are also time and personnel consuming. Moreover, to date no defined cutoff value exists that distinguishes between normal and pathological FS-VOR. We therefore tested the FS-VOR in healthy controls and patients with pathological FS-VOR. We then developed a bedside test that allows for fast, easy to interpret and reliable assessment of FS-VOR and enables video documentation of the results.

## Methods

### Ethical standard

The study was approved by the central ethics committee of the Brandenburg Medical Council (S10(a)/2015) and has therefore been performed in accordance with the ethical standards laid down in the 1964 Declaration of Helsinki and its later amendments and is listed in the German Trials Register (DRKS00013968). Participants provided written informed consent prior to their inclusion into the study.

### Participants

Between 2015 and 2017, 20 healthy subject (14 women, age 56 ± 15) and 20 patients with cerebellar disorders (10 women, age 70 ± 11 years, 12 probable MSA, 3 cerebellar ischemia, 1 episodic cerebellar ataxia, 1 spinocerebellar ataxia, 1 paraneoplastic cerebellar degeneration, and 2 sporadic cerebellar degeneration) were included in the study after providing written informed consent. One screened patient with probable MSA did not show pathological FS-VOR or other cerebellar pathology, was diagnosed with MSA of the Parkinsonian variant and excluded. Both groups underwent VNG and smartphone bedside testing.

### Video-nystagmography

VNG was performed as described previously [6] and adapted as follows: patients were seated on a swivel chair and rotated (duration 30 s amplitude 90°, frequency 0.83 Hz). A 90° angle was marked on the floor, and patients were rotated by hand from side to side at a peak to peak frequency of 50 bpm with the aid of a metronome, thereby achieving a peak velocity 118°/s. The horizontal ocular movements were recorded using a head-mounted VNG mask (VO25, Interacoustics, Denmark). For VOR recording, the mask was shut to disable visual fixation during rotation. For FS-VOR assessment, the mask was opened and the subject asked to fixate an extended thumb of their outstretched arm during rotation. Nystagmus beats, horizontal eye position and maximum horizontal slow phase velocity (SPV) were recorded.

### Smartphone bedside test

For the smartphone bedside test, patients were seated on a swivel chair and rotated in an identical way (vid.1). Patients were asked to hold a smartphone (iPhone 6S, Apple Inc., CA, USA) at arm's length and to fixate the lens. Eye movements were recorded for 30 s during rotation.

### Video rating

FS-VOR videos of the bedside test were assessed by two medical nurses, two neurology residents, and two consultant neurologists and movement disorders specialists, all blinded to the condition of the participants. The raters dichotomously rated the videos as either pathological or normal. The latter required complete or near-complete nystagmus suppression during FS-VOR testing.

### Data analysis

Data were analyzed utilizing Real Statistics Resource Pack software version 5.9.2. Distribution was calculated applying the Shapiro–Wilk test, and variance using the *F* test. Normally distributed values of homogenous variance were compared using the non-paired parametric *t* test, and not normally distributed values using the Mann–Whitney *U* test.

Fixation suppression ability was quantified as percentage of the reduction of nystagmus beats or nystagmus slow phase velocity from VOR testing to FS-VOR testing. Negative values were set to zero.

Results were compared to the video ratings. Inter-test reliability between VNG and video analysis was expressed by Cohen's kappa and inter-rater reliability by Fleiss' kappa. Sensitivity and specificity were calculated. Rater-specific ROC analyses were performed and AUC values calculated.

## Results

### Video-nystagmography and cutoff value for FS-VOR

**Fig. 1:**
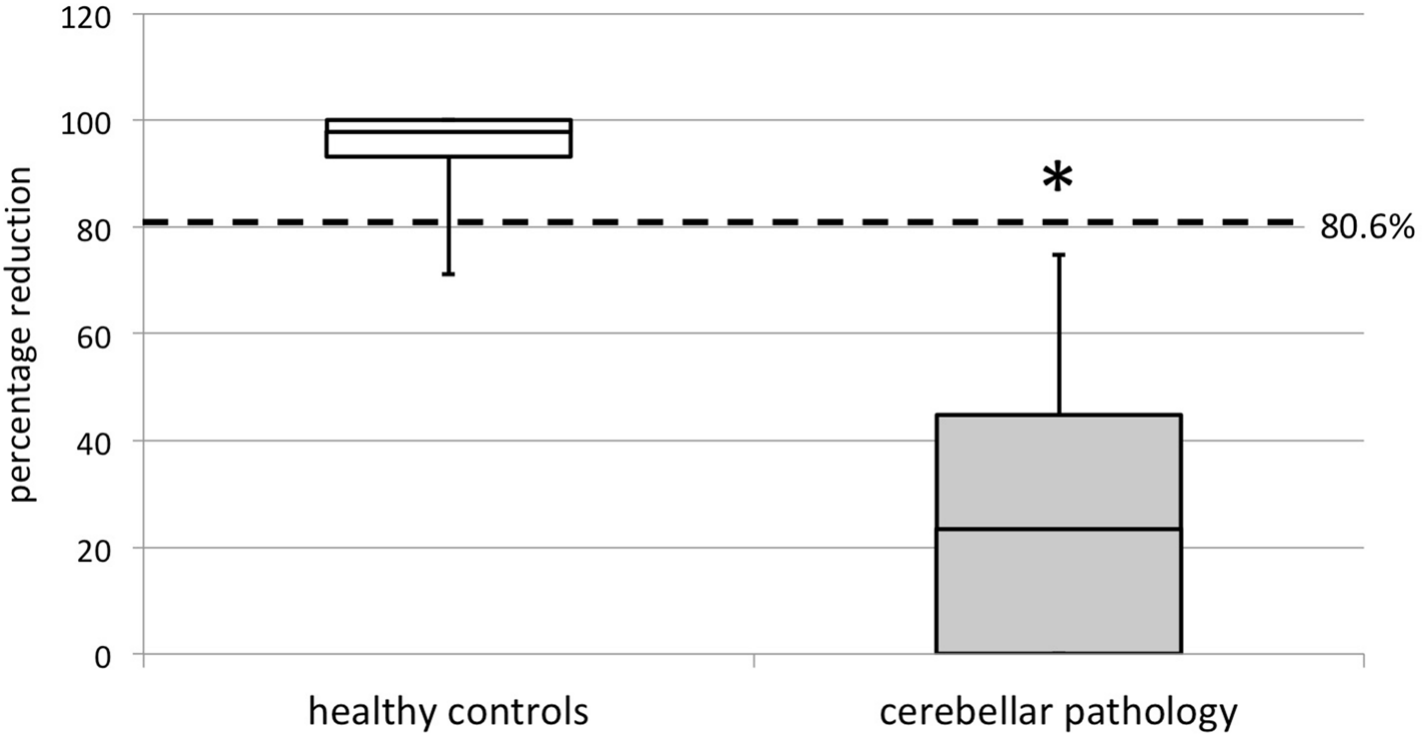
Percentage of nystagmus beats reduction during FS-VOR in healthy controls and patients with cerebellar pathology. Healthy controls 97.9% [93.2–100.0], cerebellar pathology 23.4% [0.0–44.8] (median [IQR]; *p < 0.0001). The dotted line represents the cutoff level at 80.6% reduction of nystagmus beats, corresponding to the mean − 2 SD or the 2.5th percentile of the normal distribution of the healthy cohort

**Fig. 2:**
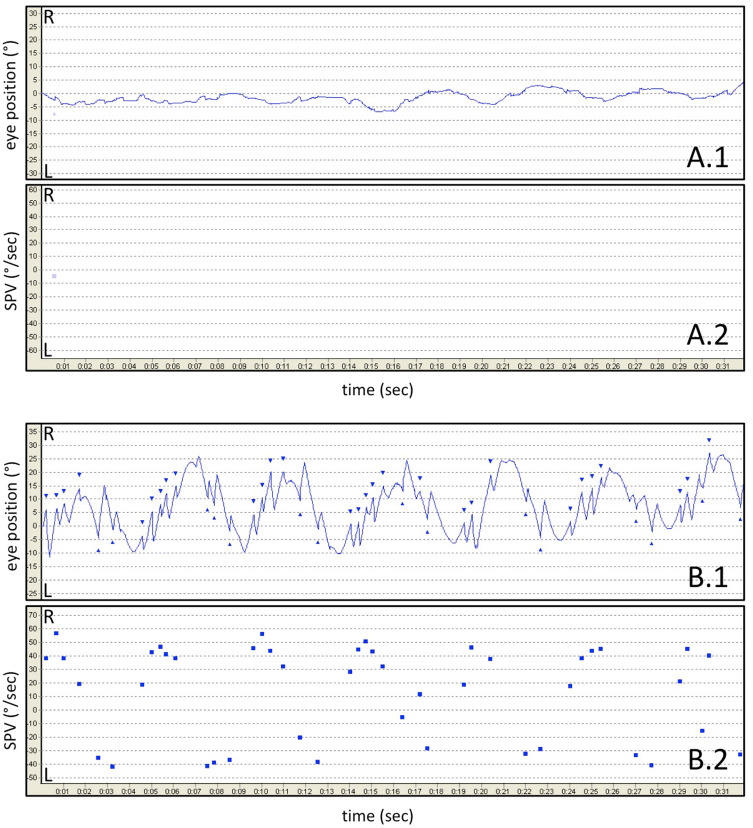
Video-nystagmography of FS-VOR in one healthy subject (**a**) and one patient with cerebellar pathology (**b**), assessing the horizontal eye position (1) and horizontal slow phase velocity (SPV) (2)

During VNG, VOR could be provoked both in healthy controls and patients with cerebellar pathology. Cerebellar patients showed more nystagmus beats and faster maximum SPV (Table 1). When performing FS-VOR during VNG, healthy controls were able to nearly completely suppress the VOR by mean ± SD [range] 95.0% ± 7.2 [71.1—100%] (*p* < 0.0001; Fig. 1; example Fig. 2). Cerebellar patients were less capable of reducing nystagmus beats when performing FS-VOR and achieved a reduction of only 26.3% ± 25.1 [0—74.7%] (*p* = 0.02), indicative of FS-VOR failure in comparison to healthy controls (*p* < 0.0001, Table 1A; Fig. 1; example Fig. 2). When assessing the maximum SPV, a nystagmus beat reduction of 93.0% ± 8.1 was calculated for healthy controls in contrast to 42.4 ± 27.6 (*p* < 0.0001) in cerebellar patients (Table 1B). Moreover, cerebellar patients had significantly more nystagmus beats and higher maximum slow phase velocities than healthy controls (Table 1, example Fig. 2).

**Table 1:** Absolute values and percentage reduction of (A) nystagmus counts and (B) maximum SPV during VOR and FS-VOR assessment in healthy controls and patients with a cerebellar syndrome. Values are indicated as mean ± SD

	VOR	FS-VOR	%Red	*p* values
(A) Nystagmus beats				
Healthy controls (*n* = 20)	47.3 ± 19.8	2.5 ± 3.6	95.0 ± 7.2	2.6 × 10^–8^
Cerebellar syndrome (*n* = 19)	63.5 ± 21.5	47.1 ± 22.7	26.1 ± 25.1	0.02
*p* values	0.006	4.8 × 10^–8^	9.7 × 10^–8^	
(B) Maximum SPV				
Healthy controls (*n* = 20)	38.5 ± 12.2	2.7 ± 3.3	93.0 ± 8.1	3.1 × 10^–8^
Cerebellar syndrome (*n* = 19)	50.3 ± 9.5	31.3 ± 20.3	42.4 ± 27.6	0.0005
*p* values	0.0007	1.2 × 10^–7^	2.7 × 10^–7^

The cutoff value separating normal and pathological FS-VOR was determined as − 2 SD of nystagmus beat reduction, equivalent to the 2.5th percentile of the normal distribution of our healthy cohort and set at 80.6% (Fig. 1, dotted line).

### Bedside test and video ratings

Six raters rated the bedside test videos of the 20 healthy controls (120 ratings) and the 19 cerebellar patients (114 ratings) (video1). 113/114 (99.1%) of the pathological and 110/120 (91.7%) of the normal FS-VOR videos were identified correctly. Video raters achieved an inter-rater reliability of *k* = 0.85 [0.76–0.93] (Fleiss' kappa [95% CI]). The sensitivity of the video ratings to detect an impaired FS-VOR was 99% and its specificity 92%.

When comparing the video ratings with the VNG results, inter-test agreement was *k* = 0.91 [0.84–0.96] (Cohen's kappa [95% CI]). Rater-specific ROC analyses revealed AUC values ranging from 0.934 to 0.997.

## Discussion

Video-nystagmography revealed that patients with a cerebellar syndrome showed significantly increased nystagmus beats and slow phase velocities during VOR testing in comparison to healthy controls. These results are in line with previous findings [7] and indicative of a release of vestibular nuclei activity from cerebellar inhibition due to cerebellar pathology [8, 9]. However, a defined cutoff value has to our knowledge not yet been defined, and we for the first time report a distribution-based threshold delineating normal from pathological fixation suppression of the vestibulo-ocular reflex (FS-VOR): during sinusoidal rotation, healthy controls were able to reduce nystagmus beats during FS-VOR by 95.0% ± 7.2 (*p* < 0.0001). In contrast, cerebellar patients largely failed to suppress the VOR during fixation, with a nystagmus beat reduction of only 26.3% ± 25.1 (*p* = 0.02). The cutoff value dividing normal from pathological FS-VOR was set at 80.6% reduction of nystagmus beats, corresponding to − 2 SD from the mean or the 2.5th percentile of the normal distribution of our healthy cohort.

The blinded video ratings of the smartphone bedside test showed an inter-rater reliability of *k* = 0.85 [0.76–0.93], expressing high accordance among raters, regardless of their medical degree and qualification. Furthermore, there was an excellent inter-test reliability with *k* = 0.91 [0.84–0.96]. The sensitivity of the video ratings of 99% and the specificity of 92% suggest that this bedside test is a useful tool to detect an impaired FS-VOR.

Our proposed test has obvious advantages when compared with VNG: (1) today almost everyone owns a smartphone to perform the test, (2) swivel chairs are available everywhere, (3) the test is easily performed and (4) has a high sensitivity and specificity.

Undoubtedly, the sample size is small, and further studies in a larger cohort should verify these findings.

While performing the test, the song "Nothing Else Matters" (by Metallica, composed by James Hetfield and Lars Ulrich) can be used as a mnemonic for two purposes: firstly, the patient is advised to fixate the smartphone's lens to maintain fixation, hence "Nothing Else Matters". Secondly, the song is recorded at 50 bpm, the speed at which the 90° chair rotation must be performed to achieve the peak velocity of 118°/s.

## Electronic supplementary material

Below is the link to the electronic supplementary material.

Video 1: Assessment of fixation suppression of the vestibulo-ocular reflex in one healthy control and one patient with cerebellar pathology. (MOV 7672 kb)

## Data Availability

Anonymized data will be shared by request from any qualified investigator.

## References

[1] Dichgans J, von Reutern GM, Römmelt U (1978). Impaired suppression of vestibular nystagmus by fixation
in cerebellar and noncerebellar patients. Arch Psychiatr Nervenkr.

[2] Halmagyi GM, Gresty MA (1979). Clinical signs of visual-vestibular
interaction. J Neurol Neurosurg Psychiatry.

[3] Gauthier GM, Vercher JL (1990). Visual vestibular interaction: vestibulo-ocular reflex
suppression with head-fixed target fixation. Exp Brain Res.

[4] Strupp M, Kremmyda O, Adamczyk C (2014). Central ocular motor disorders, including gaze palsy and
nystagmus. J Neurol.

[5] Anderson T, Luxon L, Quinn N (2008). Oculomotor function in multiple system atrophy: clinical
and laboratory features in 30 patients. Mov Disord.

[6] Blödow A, Heinze M, Bloching MB (2014). Caloric stimulation and video-head impulse testing in
Ménière’s disease and vestibular migraine. Acta Otolaryngol.

[7] Shaikh AG, Marti S, Tarnutzer AA (2011). Ataxia telangiectasia: a “disease model” to understand
the cerebellar control of vestibular reflexes. J Neurophysiol.

[8] Walker MF, Zee DS (2005). Cerebellar disease alters the axis of the
high-acceleration vestibuloocular reflex. J Neurophysiol.

[9] Walker MF, Zee DS (1999) Directional abnormalities of vestibular and optokinetic responses in cerebellar disease. Ann N Y Acad Sci 871:205–20. https://www.ncbi.nlm.nih.gov/pubmed/10372073. Accessed 25 Feb 2019 10.1111/j.1749-6632.1999.tb09186.x 10372073

