# Correction to: Diagnostic accuracy of a smartphone bedside test to assess the fixation suppression of the vestibulo‑ocular reflex: when nothing else matters

**DOI:** 10.1007/s00415-020-10035-x

**Published:** 2020-07-10

**Authors:** Florin Gandor, Manfred Tesch, Hannelore Neuhauser, Doreen Gruber, Hans-Jochen Heinze, Georg Ebersbach, Thomas Lempert

**Affiliations:** 1 Movement Disorders Hospital, Kliniken Beelitz GmbH, Strasse nach Fichtenwalde 16, 14547 Beelitz-Heilstätten, Germany; 2 Department of Neurology, Otto-Von-Guericke University, Magdeburg, Germany; 3 Department of Neurology, Schlosspark-Klinik Berlin, Berlin, Germany; 4 Robert-Koch-Institut, Berlin, Germany

## Correction to: Journal of Neurology (2020) 267:2159–2163 10.1007/s00415-020-09947-5

The article Diagnostic accuracy of a smartphone bedside test to assess the fixation suppression of the vestibulo‑ocular reflex: when nothing else matters, written by Florin Gandor, Manfred Tesch, Hannelore Neuhauser, Doreen Gruber, Hans‑Jochen Heinze, Georg Ebersbach and Thomas Lempert, was originally published electronically on the publisher’s internet portal on 01 June 2020 without open access. With the author(s)’ decision to opt for Open Choice the copyright of the article changed on 29 June 2020 to © The Author(s) 2020 and the article is forthwith distributed under a Creative Commons Attribution 4.0 International License (https://creativecommons.org/licenses/by/4.0/), which permits use, sharing, adaptation, distribution and reproduction in any medium or format, as long as you give appropriate credit to the original author(s) and the source, provide a link to the Creative Commons licence, and indicate if changes were made.

The original article has been corrected.

